# Design of a highly thermotolerant, immunogenic SARS-CoV-2 spike fragment

**DOI:** 10.1074/jbc.RA120.016284

**Published:** 2020-11-23

**Authors:** Sameer Kumar Malladi, Randhir Singh, Suman Pandey, Savitha Gayathri, Kawkab Kanjo, Shahbaz Ahmed, Mohammad Suhail Khan, Parismita Kalita, Nidhi Girish, Aditya Upadhyaya, Poorvi Reddy, Ishika Pramanick, Munmun Bhasin, Shailendra Mani, Sankar Bhattacharyya, Jeswin Joseph, Karthika Thankamani, V. Stalin Raj, Somnath Dutta, Ramandeep Singh, Gautham Nadig, Raghavan Varadarajan

**Affiliations:** 1 Molecular Biophysics Unit (MBU), Indian Institute of Science, Bengaluru, India; 2 Mynvax Private Limited, ES12, Entrepreneurship Centre, SID, Indian Institute of Science, Bengaluru, India; 3 Translational Health Science and Technology Institute, NCR Biotech Science Cluster, Faridabad, India; 4 Virology Scientific Research (VSR) Laboratory, School of Biology, Indian Institute of Science Education and Research Thiruvananthapuram (IISER TVM), Kerala, India; 5 Jawaharlal Nehru Centre for Advanced Scientific Research, Bengaluru, India

**Keywords:** glycosylation, microbial, *Pichia*, thermostable, ACE2, AUC, area under the curve, CPE, cytopathic effect, DMEM, Dulbecco's Modified Dulbecco's Medium, HRP, horseradish peroxidase, IAEC, Institutional Animal Ethics committee, IMAC, immobilized metal affinity chromatography, NTD, N-terminal domain, PBS, phosphate buffered saline, PEI, polyethylenimine, RBD, receptor-binding domain, RBM, receptor binding motif, SEC, size-exclusion chromatography

## Abstract

Virtually all SARS-CoV-2 vaccines currently in clinical testing are stored in a refrigerated or frozen state prior to use. This is a major impediment to deployment in resource-poor settings. Furthermore, several of them use viral vectors or mRNA. In contrast to protein subunit vaccines, there is limited manufacturing expertise for these nucleic-acid-based modalities, especially in the developing world. Neutralizing antibodies, the clearest known correlate of protection against SARS-CoV-2, are primarily directed against the receptor-binding domain (RBD) of the viral spike protein, suggesting that a suitable RBD construct might serve as a more accessible vaccine ingredient. We describe a monomeric, glycan-engineered RBD protein fragment that is expressed at a purified yield of 214 mg/l in unoptimized, mammalian cell culture and, in contrast to a stabilized spike ectodomain, is tolerant of exposure to temperatures as high as 100 °C when lyophilized, up to 70 °C in solution and stable for over 4 weeks at 37 °C. In prime:boost guinea pig immunizations, when formulated with the MF59-like adjuvant AddaVax, the RBD derivative elicited neutralizing antibodies with an endpoint geometric mean titer of ∼415 against replicative virus, comparing favorably with several vaccine formulations currently in the clinic. These features of high yield, extreme thermotolerance, and satisfactory immunogenicity suggest that such RBD subunit vaccine formulations hold great promise to combat COVID-19.

SARS-CoV-2 is the etiological agent of the ongoing COVID-19 pandemic (1, 2). As on October 30, 2020, there are ∼44.6 million infections and ∼1.1 million deaths worldwide (3). The major surface protein of SARS-CoV-2 is the spike glycoprotein. Like several other viral surface glycoproteins, it is a homotrimer, with each protomer consisting of two subunits S1 and S2. The S1 subunit consists of an N-terminal domain (NTD), linker and receptor-binding domain (RBD), and two small subdomains SD1 and SD2 (4, 5, 6) (Fig. 1, *A*–*D*). The RBD domain of the spike glycoprotein binds to the cell surface receptor ACE2, followed by endocytosis or fusion mediated *via* the fusion peptide located on the S2 subunit (7). Most of the neutralizing antibody responses are targeted to the RBD (8, 9, 10, 11, 12, 13, 14); though very recently, neutralizing antibodies against the NTD have also been identified (15). It is thus unclear whether the full-length spike or the RBD is a better immunogen.

**Figure 1:**
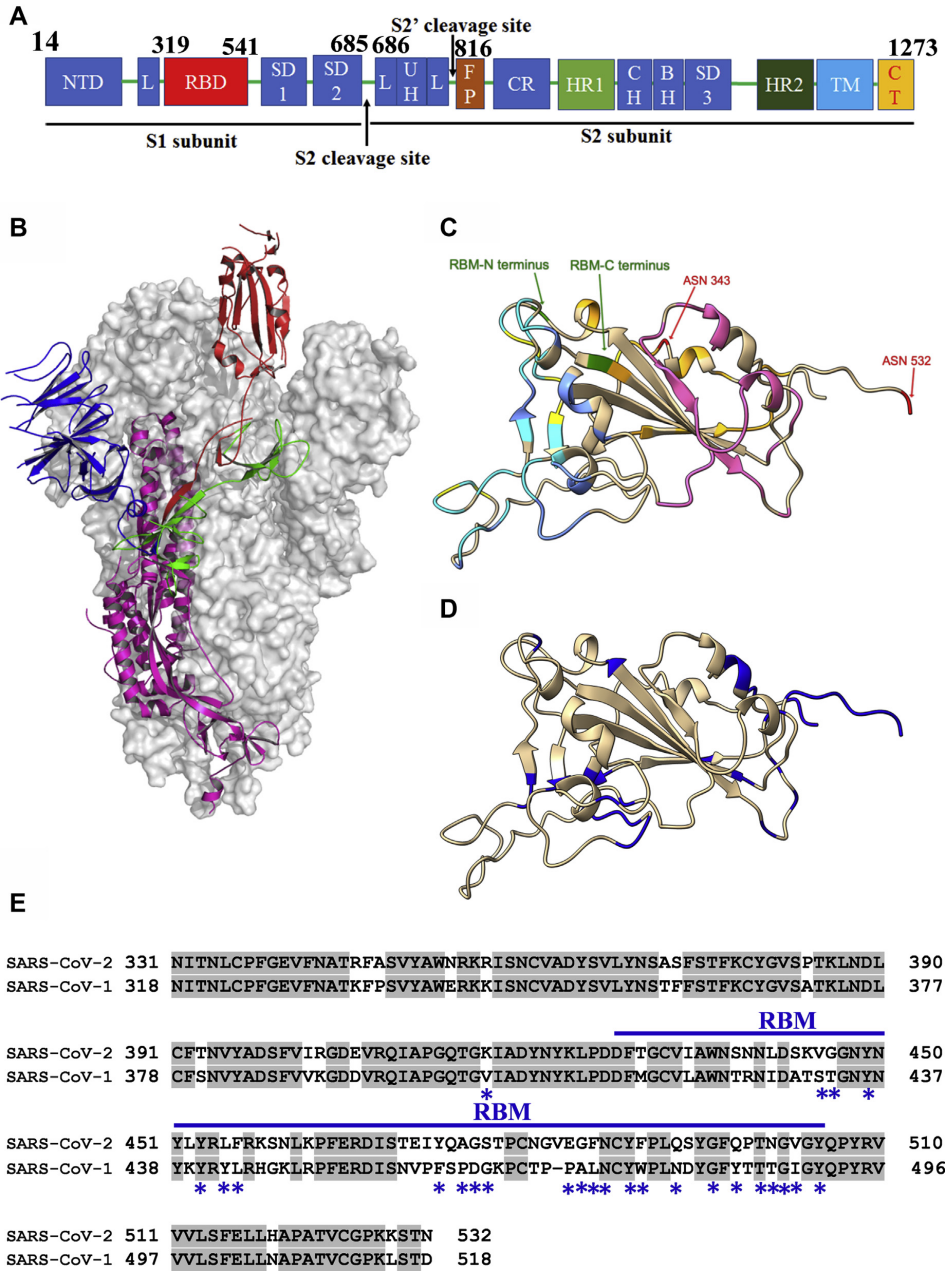
**S-protein domain organization, structure of spike and receptor-binding domain of SARS-CoV-2.***A*, linear map of the S protein spike with the following domains: NTD, N- terminal domain; L, linker region; RBD, receptor-binding domain; SD, subdomain; UH, upstream helix; FP, fusion peptide; CR, connecting region; HR, heptad repeat; CH, central helix; BH, β-hairpin; TM, transmembrane region/domain; CT, cytoplasmic tail. *B*, spike ectodomain trimer highlighting protomer with RBD in the “up” conformation, NTD in dark blue, RBD in brick red, SD1 and SD2 in green, and S2 subunit in magenta (PDB: 6VSB). *C*, epitopes for known RBD-directed neutralizing antibodies. The N and C termini of the receptor-binding motif (RBM) are labeled and in green. Residues at the binding interfaces with hACE2 are in cyan. The B38 epitope has considerable overlap with the hAce2 interface, nonoverlapping residues are in light blue. Epitopes for S309, P2B-2F6 are in orange and yellow. Epitope for CR3022 is in pink, this overlaps substantially with the potent neutralizing antibody H014. The conserved N-glycosylation site at 343 and the engineered, immune masking glycosylation site at 532 are shown in red. *D*, exposed residues with solvent accessible surface area that are not part of any neutralizing epitope identified so far are shown in dark blue. The largest such stretch is at the C-terminus where the engineered glycosylation site is placed. *E*, sequence alignment of SARS-CoV-1 (residues: 318–518) and SARS-CoV- 2 (residues 331–532), the blue line indicates the RBM, the gray highlight indicates residues conserved in both SARS-CoV-1 and SARS-CoV-2, and the blue asterisks indicate the ACE2 binding residues. (PDB: 6M0J)

Over 150 vaccine candidates are under development globally (16). Some vaccine candidates that have entered rapidly into clinical phase testing include mRNA vaccine candidates by Moderna (mRNA-1273), BioNTech (BNT162b1) (17), and CureVac (CVnCoV), a Chimpanzee Adenovirus vector vaccine by University of Oxford and AstraZeneca (ChAdOx1-S) (18), a nonreplicating adenovirus type-5 (Ad5) vaccine by Cansino (Ad5-nCoV) (19), a DNA vaccine by Inovio (INO-4800) (20), inactivated virus vaccines by Sinovac (PiCoVacc) (21) and Bharat Biotech (COVAXIN), a native-like trimeric subunit spike protein vaccine by Clover Biopharmaceuticals/GSK/Dynavax (SCB-2019), and a full-length recombinant glycoprotein nanoparticle vaccine by Novavax (NVX-CoV2373) (16, 22). The majority of the above employ full-length spike or the corresponding ectodomain as the antigen. Although there is some encouraging preclinical and Phase 1 clinical data, there is no precedent for use of mRNA or viral vectors, which are the farthest along in clinical development, in mass human vaccinations. In addition, with inactivated or attenuated virus, there are obvious safety issues that need careful attention. There are few studies that compare the relative immunogenicity of multiple vaccine candidates expressed in multiple platforms (23). Herein, we report a mammalian cell–expressed, glycan-engineered, RBD-based subunit vaccine candidate (mRBD) formulated with an MF59 equivalent adjuvant. In contrast to an equivalent *Pichia pastoris*–expressed RBD protein formulation, mRBD elicits titers of neutralizing antibodies in guinea pigs well above the levels required for protection in nonhuman primate challenge studies. mRBD expresses at eightfold higher levels and is substantially more tolerant to thermal stresses than a stabilized spike ectodomain without compromising immunogenicity and can be stored for over 4 weeks at 37 °C. These data suggest that it is a promising candidate for further clinical development.

## Results

### Design of a recombinant RBD subunit vaccine

The RBD of the spike protein is the major target of neutralizing antibodies (8, 10, 11, 12, 13, 14, 24). SARS-CoV-2 is 79.6% identical to SARS-CoV-1 sequences (25). The spike protein of SARS-CoV-2 is 80% identical to its homolog from SARS-CoV-1. The RBD of SARS-CoV-2 shares 74% amino acid sequence identity with the RBD of SARS-CoV-1. We hypothesized that a RBD subunit derivative that lacks flexible termini as well as unpaired cysteines and retains the ACE2 receptor binding site, located within the receptor binding motif (RBM, comprising residues 438–505, Fig. 1*E*) as well as the cryptic epitope recognized by the neutralizing antibody CR3022, would be a good immunogen. We selected the RBD residues based on SWISS Model structure-based modeling of SARS-CoV-2 sequence, prior to availability of any SARS-CoV-2 spike and RBD-ACE2 complex structures. The modeled structure closely resembles the recently determined experimental structures by X-ray crystallography or Cryo-EM (RMSD: 1.2 Å, with X-ray structure PDB: 6M0J) (5, 6, 26). In the X-ray structure, residues after 526 are disordered. Two RBD sequences were shortlisted consisting of residues 331 to 532 and 332 to 532 with retention (m331RBD) or deletion (mRBD/pRBD) of the native glycan at N331 for expression in mammalian and *P. pastoris* expression systems, respectively. The constructs for mammalian expression are designated as m331RBD and mRBD, and for *Pichia* expression, pRBD respectively. In the past few months, several potent neutralizing antibodies directed against the RBD have been isolated, and it currently appears that virtually the entire exposed surface of the RBD is targeted by neutralizing antibodies, with the exception of the C-terminal region distal from the RBM. We have introduced a glycosylation site at N532 in all the above RBD constructs to mask this region of the surface (Fig. 1, *C*–*D*).

### RBD (332–532) is more highly expressed and thermotolerant than a stabilized spike ectodomain

**Figure 2:**
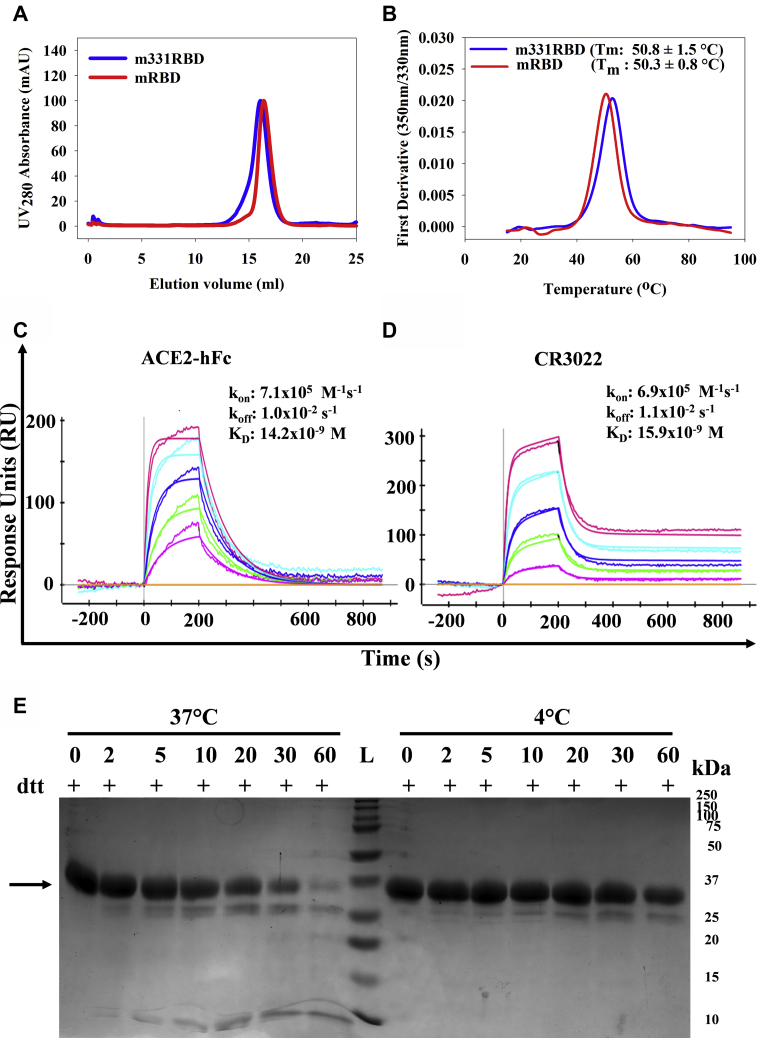
**Characterization of mammalian cell–expressed RBD.***A*, size-exclusion chromatography profile of m331RBD, mRBD immunogens with predominantly monomeric peak at ∼16.0 and ∼16.3 ml respectively on S200 10/300GL column calibrated with Biorad gel filtration marker (Cat. No. 1511901) run at flowrate of 0.5 ml/min with PBS (pH 7.4) as mobile phase. *B*, nanoDSF equilibrium thermal unfolding of m331RBD and mRBD. *C*, SPR binding sensorgrams to ACE2 receptor. The concentrations of mRBD used as analytes are 100 nM, 50 nM, 25 nM, 12.5 nM, 6.25 nM. *D*, SPR binding sensorgrams of mRBD with the neutralizing antibody CR3022. mRBD analyte concentrations are 50 nM, 25 nM, 12.5 nM, 6.2 nM, and 3.1 nM. *E*, limited proteolysis of purified mRBD protein by TPCK-treated trypsin (RBD:TPCK Trypsin = 50:1) at 4 and 37 °C.

Mammalian cell–expressed m331RBD and mRBD were purified by single step Ni-metal affinity chromatography from transiently transfected Expi293F culture supernatants. The proteins were confirmed to be predominantly monomeric by size-exclusion chromatography (SEC) (Fig. 2*A*). Proteins from both the constructs were pure and were expressed at yields of ∼68 ± 10 mg/l and ∼214 ± 9 mg/l for m331RBD and mRBD, respectively. Removal of the N-terminal glycan in m331RBD by introducing the T333H mutation resulted in substantially increased expression, similar to that of mRBD, confirming that the presence of the N-terminal glycan is responsible for reduced yield, as has been observed previously for SARS-CoV-1 RBD (27). All proteins were monomeric. In SEC, m331RBD, which has an additional glycan, elutes before mRBD (Fig. 2*A*). Given the higher yield of mRBD, most subsequent studies were carried out with this RBD derivative. nanoDSF thermal melt studies demonstrated that removal of the N-terminal glycan did not affect protein stability (Fig. 2*B*). mRBD bound ACE2-hFc with a K_D_ of about ∼14.2 nM (Fig. 2*C*) and the neutralizing antibody CR3022 with a K_D_ of 16 nM, confirming that the molecule is properly folded (Fig. 2*D*). mRBD is digested by trypsin with approximate half-lives of 20 and 60 min at 37 and 4 °C (Fig. 2*E*), respectively. The digestion kinetics is unaffected by storage for over a week at 4 °C.

A construct with identical amino acid sequence to mRBD (pRBD) was expressed and purified from *P. pastoris* strain *X-33* from a stably integrated gene cassette at a yield of ∼50 mg/l in shake flasks. The *Pichia* protein is more heterogeneous, extensively glycosylated and elutes at higher molecular weight than mRBD in both SDS-PAGE and SEC (Fig. S1, *A* and *F*). The thermal stability of the *Pichia*-purified immunogen pRBD (T_m_: 49.2 °C) is similar to mammalian cell–expressed versions (Fig. S1*B*). The protein bound with comparable affinity to ACE2-hFc and CR3022 with K_D_’s of approximately 23 nM and 30 nM, respectively, similar but slightly higher than corresponding values for mRBD (Fig. S1, *D* and *E*). *Pichia*-expressed RBD was similarly stable to thermal stress and proteolysis (Fig. S1*C*, *F* and *G*). We also attempted to express the protein in *E. coli*. The protein expressed well but was targeted to inclusion bodies. Despite multiple attempts employing a variety of refolding strategies, we were unable to obtain significant quantities of properly refolded protein, competent to bind ACE2 from *E. coli*.

**Figure 3:**
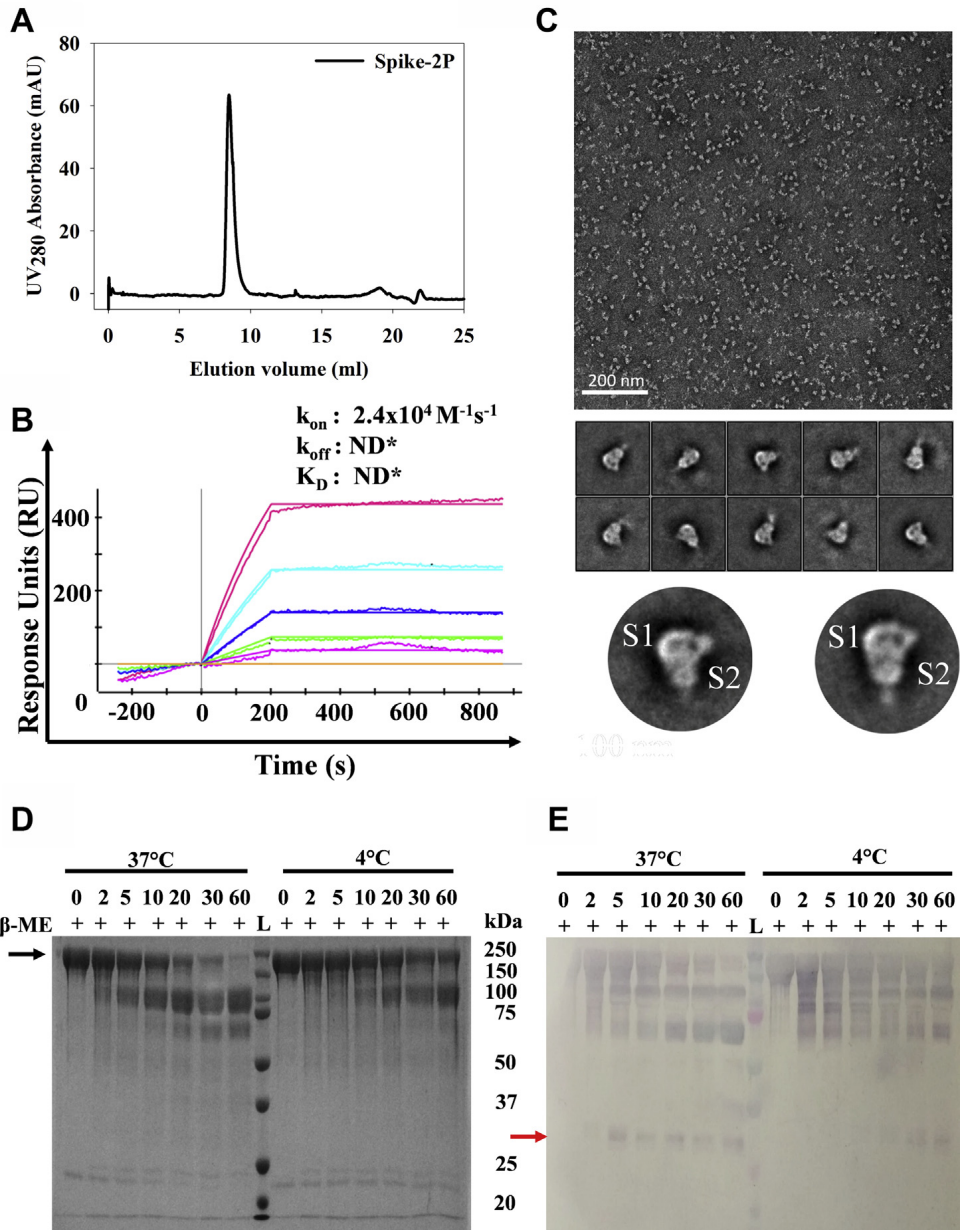
**Characterization of mammalian cell–expressed Spike-2P.***A*, size-exclusion chromatography profile of Spike-2P ectodomain with a trimeric peak at ∼8.9 ml on S200 10/300GL column calibrated with Biorad gel filtration marker (Cat. No. 1511901) run at flowrate of 0.75 ml/min with PBS (pH 7.4) as mobile phase. *B*, SPR binding sensorgrams of *Expi293F*-purified Spike-2P with immobilized ACE2-hFc. The concentrations of Spike-2P analyte used are 146 nM, 73 nM, 36.5 nM, 18 nM, 9 nM. *C*, negative-staining EM images of Spike-2P protein. TEM images indicate that the sample is homogeneous and monodisperse. Representative 2D reference-free class averages of Spike-2P protein. Well-defined class averages indicate that the Spike-2P sample has a stable and ordered structure, and enlarged views of two class averages show the S1 and S2 subunits of spike protein. *D*, SDS-PAGE Coomassie-stained gel following limited proteolysis of purified Spike-2P by TPCK-treated trypsin (RBD:TPCK Trypsin = 50:1) at 4 and 37 °C. *E*, western blot following limited proteolysis of purified Spike-2P by TPCK-treated trypsin (RBD:TPCK Trypsin = 50:1) at 4 and 37 °C, probed by α-mRBD guinea pig sera. The red arrow denotes the expected position of the RBD.

The spike ectodomain and full-length spike formulations are important SARS-CoV-2 vaccine candidates (5, 6, 22), and it is therefore important to compare mRBD with these. We purified the Spike-2P stabilized ectodomain sSpike containing mutations K968P and V969P) protein from Expi293F cells by single-step nickel chelate affinity chromatography followed by tag removal with a purified yield of ∼25 mg/l culture (5). The purified protein was observed to be trimeric on SEC and bound tightly to ACE2-hFc with little dissociation (Fig. 3, *A*–*B*). Negative-stain EM confirmed that Spike-2P purified by us adopts a native-like elongated trimeric structure (Fig. 3*C*) consistent with available structures determined by Cryo-EM (5, 6, 15). Spike-2P was rapidly digested by trypsin with approximate half-lives of 10 and 30 min at 37 and 4 °C, respectively (Fig. 3, *D*–*E*), yielding multiple RBD-containing fragments.

**Figure 4:**
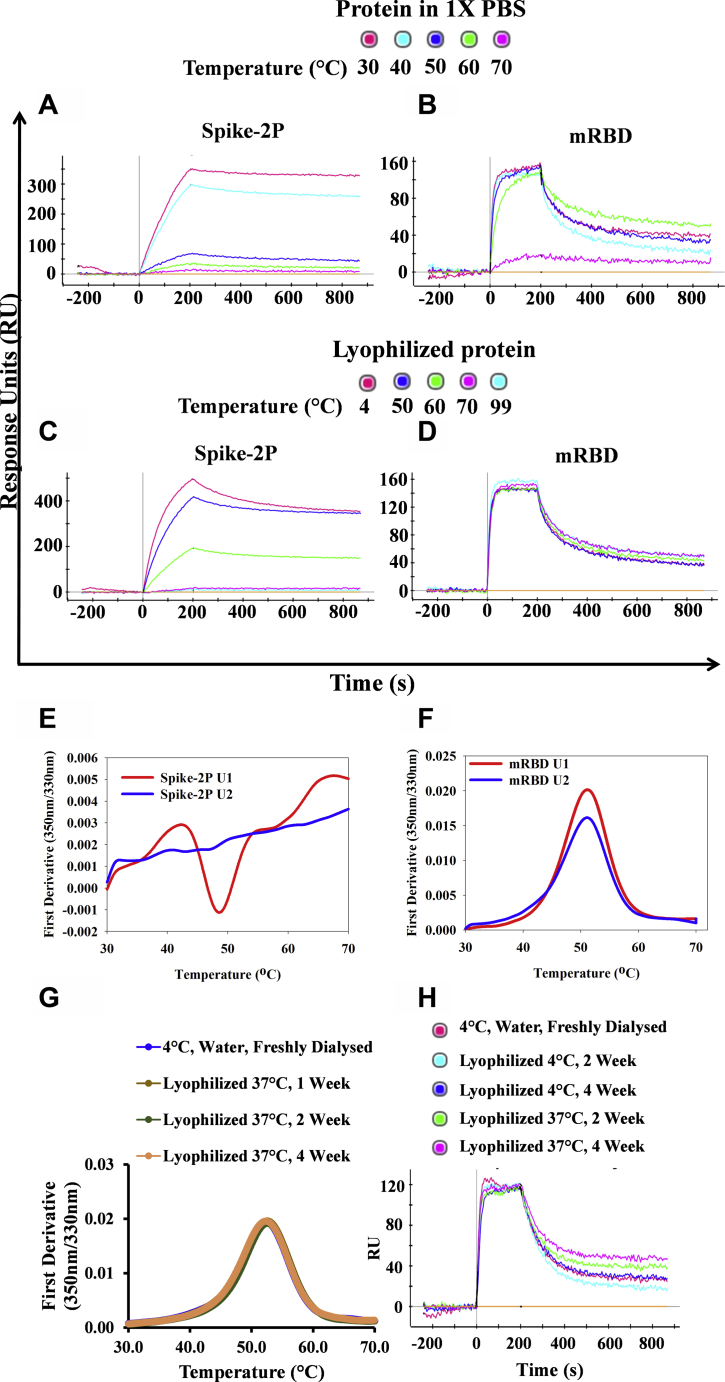
**RBD and spike protein functionality upon subjecting to thermal stress.** SPR sensorgrams of ACE2 binding by *A*–*B* protein in 1× PBS, subjected to thermal stress for 60 min. *C*–*D*, lyophilized protein subjected to thermal stress for 90 min. 100 nM of Spike-2P and mRBD were used as analytes. *E*–*F*, equilibrium thermal unfolding measured using nanoDSF for Spike-2P and mRBD. The initial and repeat unfolding scans are in red (U1) and blue (U2) respectively. *G*, equilibrium thermal unfolding measured using nanoDSF for lyophilized mRBD subjected to 37 °C incubation for up to 4 weeks. *H*, SPR sensorgrams of ACE2 binding by lyophilized mRBD incubated at 4 °C, 37 °C for up to 4 weeks. 100 nM of mRBD in 1× PBS was used as analyte. PBS, phosphate buffered saline.

Maintaining a proper cold chain during mass vaccination programs can be challenging in low- and middle-income countries (28, 29). The aggregation state of mRBD and Spike-2P proteins was unchanged upon storage at 4°C, freeze–thaw, and hour-long 37 °C incubation (Fig. S2, *A*–*B*). mRBD and spike were also incubated at various temperatures both in phosphate buffered saline (PBS) for 60 min and for 90 min in the lyophilized state. Protein conformational integrity was then assessed in ACE2 binding experiments using SPR. In PBS, mRBD is 20 °C more stable to thermal stress compared with Spike-2P (Fig. 4, *A*–*B*). Remarkably, lyophilized mRBD was stable to exposure of temperatures as high as 100 °C, whereas Spike-2P rapidly lost activity at temperatures above 50 °C both in solution and in the lyophilized state (Fig. 4, *C*–*D*). In solution, mRBD thermal unfolding was highly reversible in contrast to Spike-2P, as assessed by repetitive equilibrium thermal unfolding (Fig. 4, *E*–*F*). mRBD had identical thermal stability profiles before and after lyophilization (Fig. 2*C*). mRBD was also resistant to longer time (16 h) thermal stress and showed only small changes in the thermal unfolding profile when incubated for this time at temperatures up to 70 °C in the lyophilized state and up to 37 °C in pH 7.0 buffer (Fig. 4, *G*–*H*, Fig. S2, *D*–*E*). Even after storage at 37 °C for 4 weeks in the lyophilized state, the thermal stability as well as the Ace2 binding of the protein was unaffected (Fig. 4, *G*–*H*).

### AddaVax adjuvanted RBD elicits neutralizing antibodies in guinea pigs, functionally blocking the receptor binding motif

Guinea pigs are a widely used, outbred animal model for respiratory infectious diseases and display disease susceptibility and immune responses that are more similar to humans than the mouse model (30). Guinea pigs have also been used to evaluate other COVID-19 vaccine candidates (20, 31, 32). Guinea pigs were immunized with mammalian cell–expressed mRBD protein adjuvanted with AddaVax. AddaVax is a squalene-based oil-in-water emulsion that is a mimetic of MF59. MF59 has an extensive safety record and has been used in millions of people in the context of adjuvanted influenza vaccines (33). Animals were primed at day 0 and boosted at day 21 with bleeds at days 1 (prebleed), 14, and 35.

**Figure 5:**
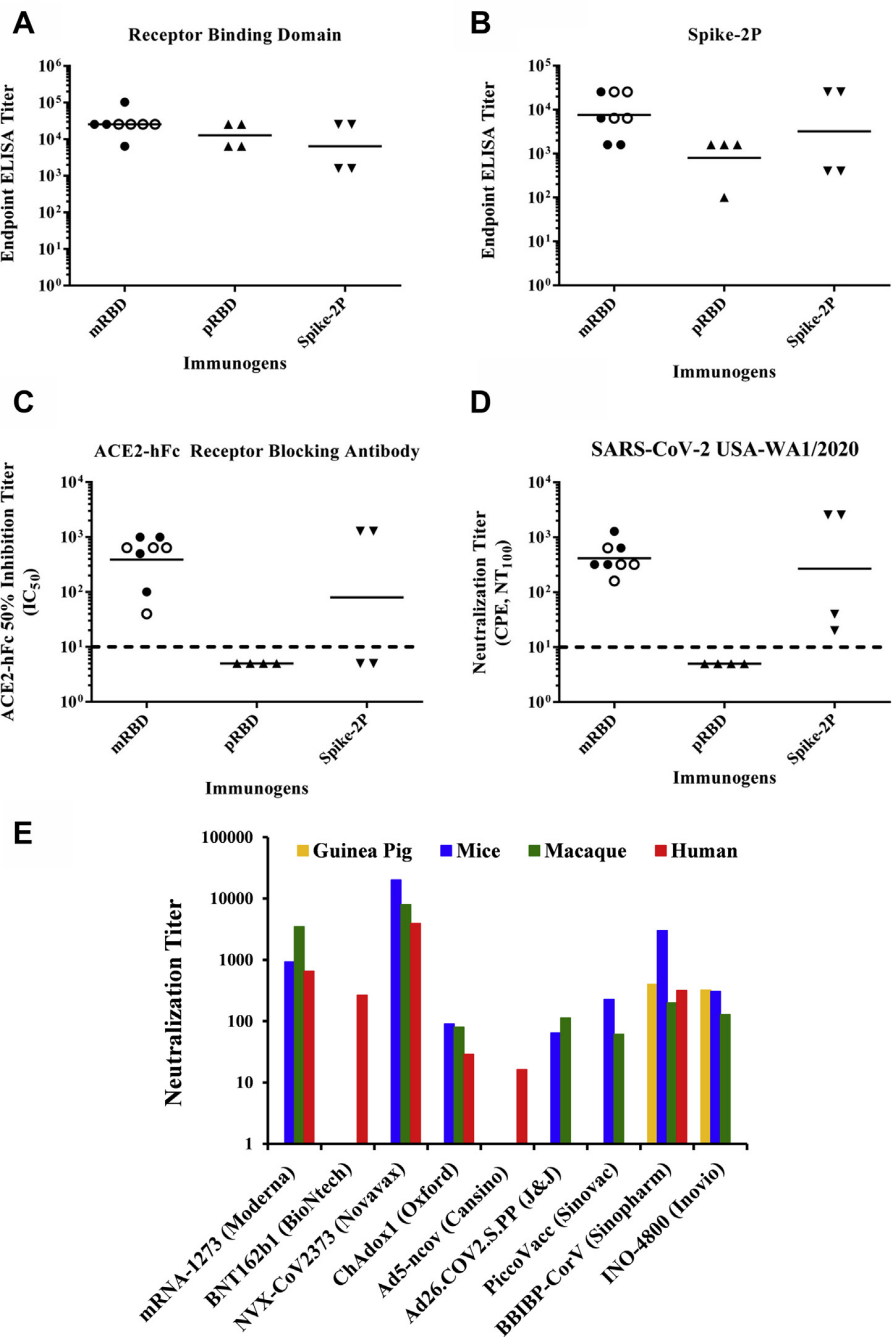
**Comparative immunogenicity data.***A*–*D*, guinea pig serum titers obtained after two immunizations with AddaVax formulated immunogens. *A*–*B*, ELISA end-point titer against mammalian cell–expressed RBD and spike ectodomain respectively. *C*, fifty percent inhibitory titers of ACE2 receptor competing antibodies from animals immunized with mRBD, pRBD and Spike-2P, respectively. Competition titers below 10 are uniformly assigned a value of 5. The dashed line represents the value 10. *D*, end-point neutralization titers in a cytopathic effect (CPE) assay against infectious SARS-CoV-2, Isolate USA-WA1/2020. The dashed line represents the value 10. (•)Sera from the first batch of animals immunized sera with mRBD, (○) second batch of animals immunized with mRBD, (▲) animals immunized with pRBD, (▼) immunized with mammalian expressed Spike-2P. *E*, live virus neutralization titers for various vaccine candidates in mice, macaques, and humans. mRNA-1273: mRNA vaccine expressing full-length Spike-2P protein assayed by PRNT (36, 37, 39). BNT162b1: nucleoside-modified mRNA vaccine expressing RBD subunit fused to T4 Fibritin-derived Foldon trimerization domain assayed by PRNT(17). NVX- CoV2373: Full-length Spike-2P adjuvanted protein vaccine assayed by CPE (22, 56). ChAdOx1 nCoV-19: Replication-deficient Chimpanzee Adenovirus vector expressing spike protein assayed by PRNT and Marburg VN(18, 38). Ad5-ncov: Replication-defective Adenovirus type 5 vector expressing spike protein assayed by CPE(19). Ad26.COV2.S: Replication-defective Adenovirus type 26 vector expressing spike protein assayed by PRNT (40, 57). PiccoVacc: Chemically inactivated SARS-CoV-2 virus vaccine assayed by CPE (21). BBIBP: Chemically inactivated SARS-CoV-2 virus vaccine assayed by CPE (31, 41). INO-4800: DNA vaccine expressing full-length Spike-2P protein assayed by CPE (20). Macaque data in INO-4800 is obtained by a SARS-CoV-2 pseudovirus neutralization assay (58).

The end-point ELISA titers to self-antigen ranged from 1:6400 to 1:102,400 after the second immunization in individual animals (Fig. 5*A*). To further confirm and extend these results, the study was repeated with the inclusion of two additional groups immunized with *Pichia*-expressed RBD and mammalian cell–expressed Spike-2P in addition to the mRBD (Fig. 5, *A*–*B*). Results with the mRBD were consistent in both studies (Fig. 5*A*). *Pichia*-expressed RBD was as immunogenic as the mRBD in terms of self-titers (Fig. S3, *A*–*B*), but sera reacted poorly with mRBD and Spike-2P. Several studies have now shown that ACE2 competition titers and neutralizing antibody titers are highly correlated (22, 34). Hence, serum competition assays were carried out (Fig. 5*C*). End-point neutralization titers with replicative virus were measured using cytopathic effect (CPE) as a readout for infection (Fig. 5*D*) and found to range from 160 to 1280. Surprisingly, the pRBD sera were nonneutralizing and poorly cross-reactive with mRBD and Spike-2P proteins (Fig. 5, *A*, *C* and *D*), presumably because of hyperglycosylation of the *Pichia*-expressed protein. In Spike-2P-immunized animals, titers were more variable than in mRBD-immunized animals though the difference in neutralization titers did not approach statistical significance. A potential advantage of using the spike as an immunogen is that it contains neutralization epitopes outside the RBD, including in the NTD (35). We therefore probed the Spike-2P sera for NTD titers using a mammalian cell–expressed NTD construct. However, all spike sera had NTD end-point ELISA titers less than 100. mRBD elicited serum neutralization titers that compared favorably with those observed with several vaccine modalities in a variety of different organisms including guinea pigs (Fig. 5*E*) (18, 19, 21, 36, 37, 38, 39, 40, 41). A recent study compared titers elicited by an inactivated virus vaccine formulation (BBIBP-CorV, Fig. 5*E*) in mice, rats, guinea pigs, and nonhuman primates; the data show close consistency across all the different animal models (31).

## Discussion

The majority of SARS-CoV-2 vaccine candidates currently in clinical testing use either full-length spike or the corresponding ectodomain as antigen and most involve relatively new nucleic acid or viral vector modalities that have not been tested in large-scale immunizations. There is an obvious need for highly expressed, stable, low-cost, and efficacious subunit vaccine formulations and for side-by-side comparisons of different candidates. In the present study, we characterized the comparative yield, stability, and immunogenicity of mammalian cell and *Pichia*-expressed RBD, as well as mammalian cell–expressed stabilized Spike-2P protein. All three candidates were successfully expressed, properly folded, and immunogenic. The data clearly indicate mammalian cell–expressed, glycan-engineered RBD to be the best of the three immunogens, displaying reversible thermal unfolding and exceptionally high thermal tolerance and stability to storage at 37 °C upto at least 4 weeks, a very important attribute for deployment in low-resource settings. The ability of the mRBD to elicit neutralizing antibodies was comparable with that of the spike ectodomain as also seen in a recent rabbit immunogenicity study with a different RBD derivative comprising residues 319–541 (23). The RBD fragment could also be expressed at high yield in the microbial host *P. pastoris* and was properly folded, stable, and immunogenic. Interestingly, an alhydrogel adjuvanted formulation of a related SARS-CoV-1 RBD construct was recently shown to be immunogenic and protect mice from SARS-CoV-1 challenge (42). Unfortunately, in the present study when pRBD was used as an immunogen, the elicited antibodies were poorly reactive with either the mammalian cell–expressed RBD or the corresponding spike ectodomain. Further, they failed to block binding of RBD to the ACE2 receptor, suggesting that further alterations to the *Pichia*-expressed sequence or adjuvant, use of an alternative *Pichia* strain, or optimization of growth/fermentation conditions are required before it can be used as an effective immunogen. Recently, various RBD-derived subunit vaccine candidates have been tested for immunogenicity employing varying fragment lengths, fusion adaptors (Fc, dimers), and adjuvants. No antibody-dependent enhancement of infection, immunopathologies, or Th2 bias has been observed with the SARS-CoV-2 RBD subunit derivatives examined so far (42, 43, 44, 45). Three independent studies used RBD-Fc fusions with one study using RBD residues 331 to 527, another used RBD-Fc from Sino Biologicals (residues not mentioned), and a third used a full-length S1-Fc fusion (residues 14–685) reporting viral neutralizing antibody titers of ∼100 to 400, 1280, and NT_50_ derived from pseudoviral neutralizations of 378 respectively (43, 44, 46). One study employed a week-long intraperitoneal immunization regime that is difficult to implement in large-scale human vaccination programs (46). The other studies utilizing RBD-Fc and S1-Fc (43, 44), employed Freund’s adjuvant, again not used in human vaccinations. For the present mRBD formulation, both the IC_50_ values in the ACE2 competition assay and the viral neutralization titers were about 2% of the corresponding ELISA end-point titers, suggesting that a significant fraction of the elicited antibodies are neutralizing. Oligomerization and nanoparticle display strategies have proven to induce appreciably higher neutralizing antibody titers than corresponding monomers; this could be potentially be exploited with our mRBD construct in future studies (45, 47, 48). However, the effect of these modifications, as well as the exact choice of chain termini, which differ between the various RBD constructs, on thermotolerance remains to be studied. Additionally, display on a heterologous scaffold will likely elicit significant antibody titers against the scaffold as well as the displayed immunogen, which might pose regulatory challenges. An mRNA vaccine encoding a longer RBD fragment (319–541) elicited approximately comparable neutralization titers in mice and macaques to those observed in the present study. In the same study, a corresponding luciferase reporter mRNA formulation was shown to tolerate 37 °C incubation for a week with ∼13% loss in activity (49). Multiple studies employing a variety of vaccine formulations and modalities have now demonstrated that SARS-CoV-2 viral neutralization titers in small animals, including mice and guinea pigs, are predictive of immunogenicity in macaques and humans (Fig. 5*E*) (17, 18, 19, 20, 22, 23, 36, 37, 38, 39, 42, 43, 44, 45, 50). Despite promising immunogenicity in several cases, all of the above liquid vaccine formulations were either refrigerated or frozen prior to use. In contrast, the mRBD described above can be stored lyophilized without refrigeration for at least 4 weeks and is also tolerant to transient high-temperature exposure. In future studies, the present formulation will be tested for its ability to confer protection against challenge in an appropriate model, following which it can be advanced to clinical development. It will also be valuable to examine if other RBD protein formulations with different chain termini are similarly thermotolerant.

## Experimental procedures

### SARS-CoV-2 RBD, NTD, spike ectodomain, and antibody expression constructs

Two fragments of the SARS-CoV-2 spike protein (S) (accession number YP_009724390.1) consisting of the RBD residues 331 to 532 with an N-terminal glycosylation site and 332 to 532 with deletion of the N-terminal glycan site deletion were chosen. Residue N532 was engineered to be glycosylated by introducing an NGS motif at the C-termini of the RBD into both immunogen sequences. The resulting sequences with an HRV-3C precision protease cleavage site linked to a 10xHistidine tag by GS linker were codon optimized for human cell expression under control of the *CMV* promoter with a tPA signal sequence for efficient secretion. The clones were named m331RBD (331–532) and mRBD (332–532). Identical RBD amino acid sequences to those described above, codon optimized for *P. pastoris* expression, were cloned into the *AOX1* promoter containing vector pPICZalphaA, containing a MATalpha signal sequence for efficient secretion. The resulting clones were named p331RBD (expressing RBD 331–532) and pRBD (expressing RBD 332–532). In the present study, only pRBD was utilized, based on expression data for the corresponding mammalian and insect cell expression clones. A spike NTD construct (residues 27–309 with and L296E mutation) under control of the *CMV* promoter with a tPA signal sequence was also designed. A spike construct, encoding a stabilized ectodomain with two Proline mutations (Spike-2P) optimized for mammalian cell expression, was obtained from the VRC, NIH (5). Genes for the heavy and light chain of the CR3022 antibody were obtained from Genscript (USA) and cloned into the pcDNA3.4 vector.

### Purification of recombinant proteins expressed in Expi293F cells

Transfections were performed according to the manufacturer’s guidelines (Gibco, Thermo Fisher). Briefly, 1 day prior to transfection, cells were passaged at a density of 2 × 10^6^ cells/ml. On the day of transfection, cells were diluted to 3.0 × 10^6^ cells/ml. Desired plasmids (1 μg plasmid per 1 ml of Expi293F cells) were complexed with ExpiFectamine293 (2.7 μl of ExpiFectamine293 per 1 μg of plasmid) and transiently transfected into Expi293F cells. Post 16 h, Enhancer 1 and Enhancer 2 were added according to the manufacturer’s protocol. Five days posttransfection, culture supernatant was collected, proteins were affinity purified by immobilized metal affinity chromatography (IMAC) using Ni Sepharose 6 Fast flow resin (GE Healthcare). Supernatant was twofold diluted with 1× PBS (pH 7.4) bound to a column equilibrated with PBS (pH 7.4). A ten-column volume wash of 1× PBS (pH 7.4), supplemented with 25 mM imidazole was performed. Finally, the bound protein was eluted with a gradient of 200 to 500 mM imidazole in PBS (pH 7.4). The eluted fractions were pooled and dialyzed thrice using a 3 to 5 kDa (MWCO) dialysis membrane (40 mm flat width) (Spectrum Labs) against PBS (pH 7.4). Protein concentration was determined by absorbance (A_280_) using NanoDrop2000c with the theoretical molar extinction coefficient calculated using the ProtParam tool (ExPASy).

### Purification of recombinant protein expressed in *P. pastoris*

Twenty micrograms of pRBD vector was linearized with the PmeI enzyme by incubating at 37 °C overnight (NEB, R0560). Enzyme was inactivated (65 °C, 15 min) prior to PCR purification of the linearized product (Qiagen, Germany). Ten micrograms of linearized plasmid was transformed into *P. pastoris* X-33 strain by electroporation as per the manufacturers protocol (Thermo Fisher). Transformants were selected on Zeocin containing YPDS plates at a Zeocin concentration of 2 mg/ml (Thermo Fisher Scientific) after incubation for 3 days at 30 °C.

Ten colonies from the YPDS plate were picked and screened for expression by inducing with 1% methanol, fresh methanol was added every 24 h. Shake flasks (50 ml) containing 8 ml BMMY media (pH 6.0) each were used for growing the cultures for up to 120 h maintained at 30 °C, 250 rpm. The expression levels were monitored by dot blot analysis with anti-His tag antibodies. The colony showing the highest expression level was then chosen for large-scale expression.

Larger-scale cultures were performed in shake flasks by maintaining the same volumetric ratio (flask: media) as the small-scale cultures. The expression levels were monitored every 24 h using sandwich-ELISA.

Cultures were harvested by centrifuging at 4000*g* and subsequent filtering through a 0.45 μm filter (Sartorius). The supernatant was bound to pre-equilibrated Ni Sepharose 6 Fast flow resin (GE Healthcare). The beads were washed with 1× PBS (pH 7.4), supplemented with 150 mM NaCl and 20 mM imidazole. Finally, the His tagged pRBD protein was eluted in 1× PBS (pH 7.4) supplemented with 150 mM NaCl and 300 mM imidazole. The eluted fractions were checked for purity on a SDS-PAGE. Following this, appropriate fractions were pooled and dialyzed against 1× PBS (pH 7.4) to remove imidazole.

### Purification of recombinant protein from E. coli

The *E. coli* expression construct, eInCV01R consisted of residues 331 to 532 of the RBD expressed under control of the *T7* promoter with an N-terminal His tag in the vector pET15b. eInCV01R was transformed in both *E. coli* BL21(DE3) (Novagen) and *E. coli* SHuffle T7 cells (NEB C3029H). Following cell growth in Terrific Broth and induction with 1 mM IPTG at an OD600 of 1 at either 30 or 37 °C, cells were grown for 10 h. Expression was seen in the insoluble and soluble fractions in these two strains respectively. Following cell lysis of the SHuffle cells, protein was purified using Ni-NTA chromatography with a yield of about 1 mg/l. The protein was aggregation prone and failed to bind ACE2-hFc. In the case of BL21(DE3), following cell lysis, inclusion bodies were solubilized in buffer containing 7 M Guanidine Hydrochloride and 10 mM mercaptoethanol. Protein was purified using Ni-NTA chromatography under denaturing conditions. Protein was diluted into refolding buffer containing 0.4 M L -Arginine, 100 mM Tris–HCl (pH 8.0), 2.0 mM EDTA (pH 8.0), 5.0 mM L-glutathione reduced, 0.5 mM L-glutathione oxidized, but precipitated. Refolding in the absence of redox buffer was also unsuccessful.

### Tag removal

The His tag was removed by subjecting proteins to digestion with HRV-3C protease (Protein: HRV-3C = 50:1) in PBS (pH 7.4) buffer and incubating at 4 °C, 16 h. The untagged protein (containing C-terminal sequence: LEVLFQ) was separated from the remaining tag protein and protease by IMAC using Ni Sepharose 6 Fast flow resin (GE Healthcare). The unbound tag-free protein was collected, and protein concentration was determined by absorbance (A_280_) using NanoDrop2000c with the theoretical molar extinction coefficient calculated using the ProtParam tool (ExPASy).

### SDS-PAGE and western blot analysis

SDS-PAGE was performed to estimate the purity of the recombinant proteins. Protein samples were denatured by boiling with sample buffer containing SDS, with or without DTT. For western blotting, following SDS-PAGE, proteins were electrophoretically transferred onto an Immobilon-P membrane (Millipore). After transfer, the membrane was blocked with 3% nonfat milk. The membrane was washed with PBST (1× PBS with 0.05% Tween-20) and incubated with antisera raised against mRBD in guinea pig (1:100). Following this, blot was washed and incubated with α-guinea pig ALP conjugated antibody (Sigma) at 1:5000. After washing with 1× PBST, blot was developed by BCIP/NBT liquid substrate system (Sigma).

### Size-exclusion chromatography (SEC)

A Superdex-200 10/300GL analytical gel filtration column (GE healthcare) equilibrated in 1× PBS (pH 7.4) buffer was used. SEC profiles were obtained using a Biorad NGC chromatography system. The area under the curve (AUC) was calculated using the peak integrate tool in the evaluation platform for various peaks from each run

### nanoDSF studies

Equilibrium thermal unfolding experiments of m331RBD (–10xHis tag), mRBD (–10xHis tag), pRBD (–10xHis tag), and Spike-2P were carried out using a nanoDSF (Prometheus NT.48). Two independent measurements were carried out in duplicate with 10 to 44 μM of protein in the temperature range of 15 to 95 °C at 40–80% LED power and initial discovery scan counts (350 nm) ranging between 5000 and 10,000. In all cases, lyophilized protein was redissolved in water, prior to DSF.

### SPR binding of immobilized ACE2-hFc/CR3022 to Spike-2P and RBD derivatives as analytes

ACE2-hFc and CR3022 neutralizing antibody binding studies with the various RBD derivatives purified from different expression platforms were carried out using the ProteOn XPR36 Protein Interaction Array V.3.1 from Bio-Rad. Activation of the GLM sensor chip was performed by reaction with EDC and sulfo-NHS (Sigma). Protein G (Sigma) at 10 μg/ml was coupled in the presence of 10 mM sodium acetate buffer pH 4.5 at 30 μl/min for 300 s in various channels. The Response Units for coupling Protein G were monitored till ∼3500 to 4000 RU was immobilized. Finally, the excess sulfo-NHS esters were quenched using 1 M ethanolamine. Following this, ∼1000 RU of ACE2-hFc or CR3022 was immobilized on various channels at a flow rate of 5 μg/ml for 100 s leaving one channel blank that acts as the reference channel. mRBD, pRBD, and Spike-2P were passed at a flow rate of 30 μl/min for 200 s over the chip surface, followed by a dissociation step of 600 s. A lane without any immobilization was used to monitor nonspecific binding. After each kinetic assay, the chip was regenerated in 0.1 M Glycine-HCl (pH 2.7) (in the case of the ACE2-hFc assay) and 4 M MgCl_2_ (in case of the CR3022 binding assay). The immobilization cycle was repeated prior to each kinetic binding assay in case of ACE2-hFc. Various concentrations of the mRBD (–10xHis tag) (100 nM, 50 nM, 25 nM, 12.5 nM, 6.25 nM), pRBD (–10xHis tag) (100 nM, 50 nM, 25 nM), and Spike-2P (–8xHis tag) (146 nM, 73 nM, 36.5 nM, 18.2 nM, 9.1 nM) in 1× PBST were used for binding studies. The kinetic parameters were obtained by fitting the data to a simple 1:1 Langmuir interaction model using Proteon Manager.

### SPR binding of immobilized ACE2-hFc to thermal stress subjected mammalian RBD/Spike-2P as analytes

Mammalian RBD/Spike-2P protein at concentration of 0.2 mg/ml in either 1× PBS or as lyophilized protein was subjected to thermal stress by incubation at the desired temperature in a thermal cycler for 60 or 90 min, respectively. Following this, lyophilized protein was resuspended in water, and SPR binding assay as described above was performed to assess the binding response using 100 nM of the thermally stressed protein.

### Limited proteolysis

An isothermal limited proteolysis assay was carried out for mRBD, pRBD, and Spike-2P using TPCK-Trypsin at 4 and 37 °C. Substrate proteins were dialyzed in autoclaved water (MQ) and reconstituted in the digestion buffer (50 mM Tris, 1 mM CaCl_2_ (pH 7.5)). Approximately one hundred micrograms of each protein was subjected to proteolysis with 2 μg of TPCK-trypsin (TPCK Trypsin: Vaccine candidate =1:50) incubated at two different temperatures 4 and 37 °C with equal volumes of sample drawn at various time points 0, 2, 5, 10, 20, 30, and 60 min, respectively. The reaction was quenched by SDS-PAGE loading buffer and incubation at 95 °C and analyzed by SDS-PAGE.

### Guinea pig immunizations

Groups of four, female, Hartley strain guinea pigs, (6–8 weeks old, approximately weighing 300 g) were immunized with 20 μg of purified antigen protein diluted in 50 μl PBS, (pH 7.4) and mixed with 50 μl of AddaVax adjuvant (vac-adx-10) (1:1 v/v Antigen: AddaVax ratio per animal/dose) (InvivoGen, USA). Immunizations were given by intramuscular injection on days 0 (prime) and 21 (boost). Blood was collected and serum isolated on days 2 (prebleed), 14, and 35, following the prime and boost immunization, respectively. All animal studies were approved by the Institutional Animal Ethics committee (IAEC) No. RR/IAEC/72-2019, Invivo/GP/084. Although there were lockdown-associated constraints on procurement of animals, group sizes of four animals are often used in comparative immunogenicity assessments (51).

### ELISA–serum binding antibody end-point titers

96-well plates were coated with immunized vaccine antigen and incubated for 2 h at 25 °C (4 μg/ml, in 1× PBS, 50 μl/well) under constant shaking (300 rpm) on a MixMate thermomixer (Eppendorf, USA). ACE2-hFc protein coating was used as a control for antigen immobilization. Following four washes with PBST (200 μl/well), wells were blocked with blocking solution (100 μl, 3% skimmed milk in 1× PBST) and incubated for 1 h at 25 °C, 300 rpm. Next, antisera (60 μl) starting at 1:100 dilution with fourfold serial dilutions were added, and plates were incubated for 1 h at 25 °C, 300 rpm. Three washes with 1× PBST were given (200 μl of 1× PBST/well). Following this, Rabbit ALP enzyme conjugated to anti-Guinea Pig IgG secondary antibody (diluted 1:5000 in blocking buffer) (50 μl/well) was added and incubated for 1 h at 25 °C, 300 rpm (Sigma-Aldrich). Subsequently, four washes were given (200 μl of 1× PBST/well). pNPP liquid substrate (50 μl/well) (pNPP, Sigma-Aldrich) was added, and the plate was incubated for 30 min at 37 °C, 300 rpm. Finally, the chromogenic signal was measured at 405 nm. The highest serum dilution, which had a signal above the cutoff value (0.02 O.D. at 405 nm), was considered as the end-point titer for ELISA.

### ACE2-hFc competition ELISA

96-well plates were coated with vaccine antigen and incubated overnight at 25 °C (4 μg/ml in 1× PBS, 50 μl/well) under constant shaking (300 rpm) on a MixMate thermomixer (Eppendorf, USA). Ovalbumin (4 μg/ml in 1× PBS, 50 μl/well) coating was used as negative control for mRBD immobilization. Next, four washes with 1× PBST were given (200 μl/well), and wells were blocked with blocking solution (100 μl 3% skimmed milk in 1× PBST) for 1 h at 25 °C, 300 rpm. Next, antisera (60 μl) starting at a dilution of 1:10 in blocking solution were added to sera competition wells, and blocking solution alone was added to the control wells. Samples were incubated for 1 h at 25 °C, 300 rpm, and three washes with 1× PBST were given (200 μl of 1× PBST/well). An additional blocking step was performed for 1 h with blocking solution (100 μl) incubated at 25 °C, 300 rpm. Following this, an excess of ACE2-hFc was added (60 μl at 20 μg/ml), and samples were incubated for 1 h at 25 °C, 300 rpm. Three washes were given (200 μl of PBST/well). Next, rabbit ALP enzyme conjugated to antihuman IgG secondary antibody (diluted 1:5000 in blocking buffer) (50 μl/well) was added, and samples were incubated for 1 h at 25 °C, 300 rpm (Sigma-Aldrich). Four washes were given (200 μl of PBST/well). pNPP liquid substrate (50 μl/well) was added, and the plate was incubated for 30 min at 37 °C, 300 rpm. Finally, the chromogenic signal was measured at 405 nm. The percent competition was calculated using the following equation:

% competition =[Absorbance (Control)– Absorbance (Sera Dilution)]∗100/[Absorbance (Control)].

Where, Absorbance (Control) is the Absorbance at 405 nm of ACE2-hFc protein binding to RBD in the absence of sera, Absorbance (Sera dilution) is the absorbance from wells where the serum dilution is incubated with ACE2-hFc protein and mRBD.

### Sandwich ELISA for monitoring RBD expression

Four microgram/milliliter ACE2 in 1× PBS pH 7.4 was coated onto ELISA strips (Thermo Fisher) for 1 h and then blocked with 3% BSA solution (1× PBS) for 1 h at RT. Samples were diluted in the blocking solution and incubated in the wells for 2 h at RT. The wells were incubated with anti-His Antibody (1:10,000 dilution) conjugated with Horseradish peroxidase (HRP) enzyme for 1 h at RT following which the reaction was visualized by adding 50 μl of the chromogenic substrate, TMB (Thermo Fisher). The reaction was stopped after 20 min with 50 μl of 1 M HCl, and the absorbance reading at 450 nm was obtained from an ELISA plate reader. Plates were washed with 1× PBS pH 7.4 after each step.

### Negative-staining sample preparation and visualization by transmission electron microscope

For visualization by transmission electron microscope, Spike-2P sample was prepared by conventional negative staining method. Briefly, carbon-coated Cu grids were glow discharged for 30 s, and 3.5 μl of sample (0.1 mg/ml) was incubated on the grid for 1 min. The extra sample and buffer solution was blotted out, and negative staining was performed using 1% Uranyl Acetate solution for 30 s. Freshly prepared grids were air-dried for 30 min. The negatively stained sample was visualized at room temperature using a Tecnai T12 electron microscope equipped with a LaB_6_ filament operated at 120 kV using a low electron dose. Images were recorded using a side-mounted Olympus VELITA (2KX2K) CCD camera using defocus ranging from −1.3 to −1.5 and a calibrated pixel size 2.54 Å/pixel at specimen level.

### Reference-free 2D classification using single-particle analysis

The evaluation of micrographs was done with EMAN 2.1 (52). Around 2500 particles projections were picked manually and extracted using e2boxer.py in EMAN2.1 software. 2D particle projections were binned by 2 using e2proc2d.py. Reference-free 2D classification of different projections of particle was performed using simple_prime2D of SIMPLE 2.1 software (53).

### CPE-based viral neutralization assay

Guinea pig sera after two immunizations (prime and boost) along with preimmune (negative control) samples were heat inactivated prior to the virus neutralization assay by incubating at 56 °C for 1 h. SARS-CoV-2 (Isolate: USA-WA1/2020) live virus, 100TCID_50_ in a volume of 50 μl was premixed with various dilutions of the serum and incubated at 37 °C for 1 h in a 5% CO_2_ incubator. Serial dilutions of the incubated premix of virus serum were added in duplicates into a 96-well plate containing VeroE6 cells (10^4^/well) and cultured for 48/96 h. After completion of incubation, the plates were assessed for virus-induced cytopathic effect (CPE), and the neutralization titre was considered as the highest serum dilution at which no CPE was observed under the microscope.

### Production of pseudotyped SARS-CoV-2 and pseudovirus neutralisation assay

The full-length synthetic construct of spike glycoprotein of SARS-CoV-2 (GenBank: MN908947) was synthesized from Genewiz, UK. The complete coding sequences of the spike genes of SARS–CoV-2 and SARS-CoV (GenBank: AY278491) lacking the endoplasmic retention signal sequence were amplified from either the synthetic construct or cDNA and cloned into pCAGGS expression vector (pCAGGS-SARS-2-S and pCAGGS-SARS-S). Pseudotyped coronaviruses were produced as previously described. Briefly, the plasmids pCAGGS-SARS2-S and pCAGGS-SARS-S were transiently expressed on HEK 293T cells using polyethylenimine (PEI) (Polysciences, USA). Twenty-four hours posttransfection, the cells were infected with VSVΔG/GFP virus, incubated for 1 h, and cells were washed thrice with 1× PBS and replaced with DMEM medium containing 1% FCS and antibiotics. Pseudotyped GFP expressing coronaviruses were harvested from the cell supernatant 24 hpi and concentrated using Amicon columns (Merck). Then the viruses were titrated in Vero E6 cells and stored at −80 °C. A Pseudovirus neutralization assay was performed as described elsewhere with minor modifications (54, 55). Guinea pig sera obtained after the first immunization were tested at a dilution of 1:10 to 1:80 for the presence of neutralizing antibodies to SARS-CoV-2 using pseudotyped virus. Briefly, Vero E6 cells (10,000 cells/well) were plated in a 96-well plate (Nunc, Thermo Scientific) the day before the neutralization assay. Twofold serially diluted sera were prepared in 96-well plates, starting at 1:10 dilution. Pseudotyped SARS-CoV2 was diluted in Dulbecco's Modified Dulbecco's Medium (DMEM) supplemented with 1% FBS and penicillin-streptomycin. Next, 50 μl pseudotyped SARS-CoV-2 was added in each well of plates, and the plates were incubated 37 °C for 1 h. SARS-CoV pseudotyped virus and SARS-CoV polyclonal antibodies (that cross-react with SARS-CoV-2) were used as a positive control. Subsequently, serum–pseudovirus mixtures were transferred to a plate containing Vero E6 cells for 1 h. Then the cells were washed twice with 1× PBS and once with medium, and cells were grown in fresh DMEM medium followed by incubation in a 5% CO2 environment at 37 °C for 24 h. The neutralization titer was measured by calculating the percentage of GFP positive cells in each well.

## Data availability

All the data are in the article

## Conflict of interest

A provisional patent application has been filed for the RBD formulations described in this article. S. K. M, S. A., R. V, S. P, R. S are inventors. G. N., R. V. are founders of Mynvax, and S. P., R. S., N. G., A. U., and P. R. are employees of Mynvax Private Limited.
