# Supplementary material for: Design of a highly thermotolerant, immunogenic SARS-CoV-2 spike fragment
Source: J Biol Chem. 2020 Nov 23;296:100025. doi: 10.1074/jbc.RA120.016284 (PMC7832000; doi:10.1074/jbc.RA120.016284)
Supplement: Fig. S1–S3 [file mmc1.pdf]

Supporting information

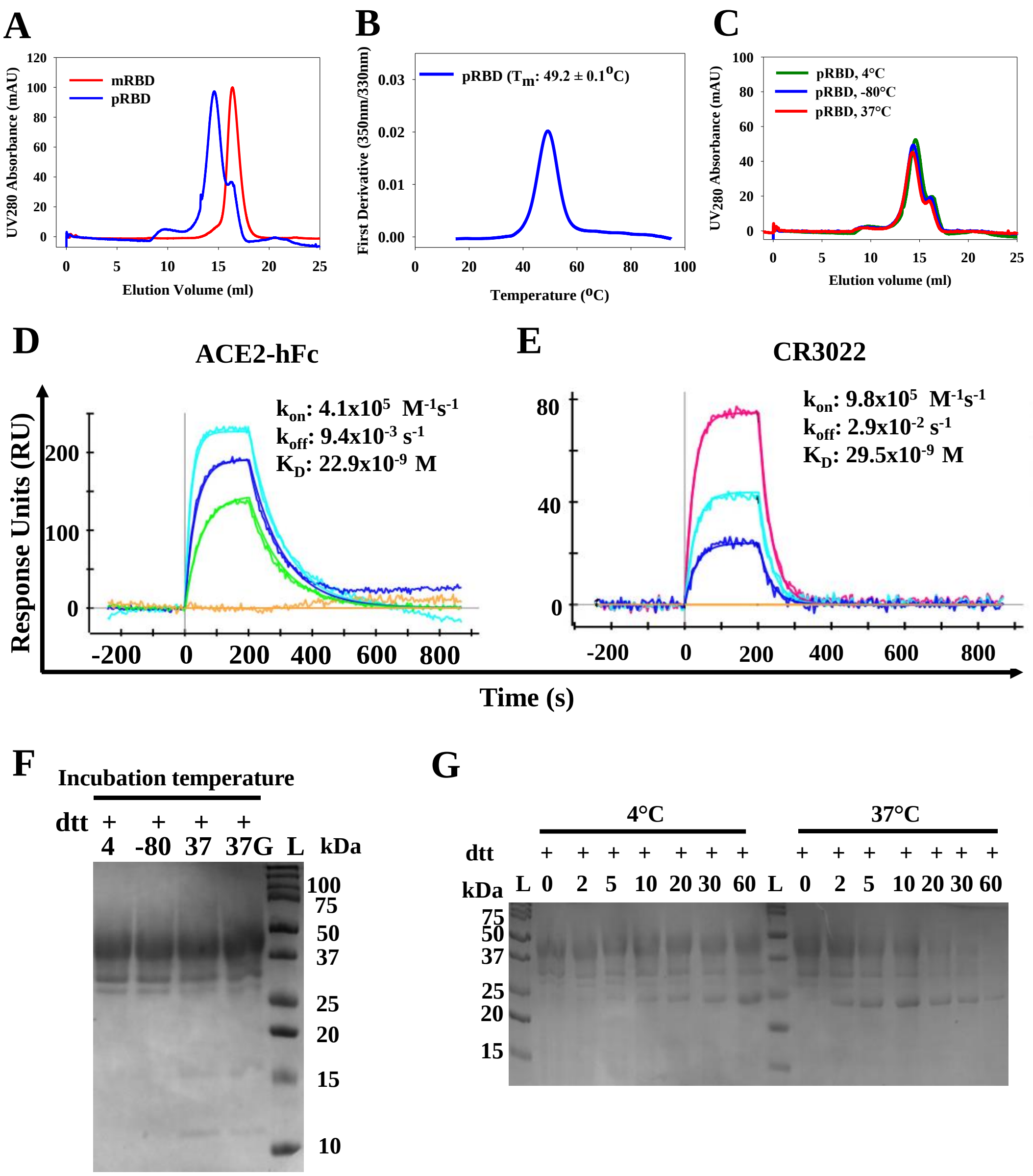

Supplementary Figure 1: Characterization of pRBD A) Comparison of SEC profiles of *Pichia* and mammalian cell expressed RBD proteins with predominantly monomeric peaks at ~14.5 mL, ~17.0 mL and ~16.3 mL respectively on an S200 10/300GL column calibrated with Biorad gel filtration marker (Cat. No. 1511901) run at flowrate of 0.5mL/min with PBS (pH 7.4) as mobile phase **B)** nanoDSF equilibrium thermal unfolding of pRBD and mRBD. **C)** Size exclusion chromatography profile of pRBD following freeze thaw, incubation at 37°C for one hour and stored overnight at 4 °C. **D)** SPR binding sensorgrams of pRBD to ACE2 receptor. The concentrations of pRBD used as analytes are 100 nM, 50 nM, 25 nM. **E)** SPR binding sensorgrams of pRBD with the neutralizing antibody, CR3022.The concentrations of pRBD used as analytes are 12.5 nM, 6.2 nM, 3.1 nM. **F)** Coomassie stained Reducing SDS-PAGE of pRBD incubated at various temperatures 4- 4°C stored protein, -80 –80 °C frozen and thawed protein, 37- protein incubated at 37°C for 1 hour without glycerol, 37G- protein incubated at 37 °C for 1 hour with 5% glycerol. **G)** Limited proteolysis of pRBD with TPCK treated trypsin (RBD:TPCK

Trypsin=50:1) at 4°C and 37°C.

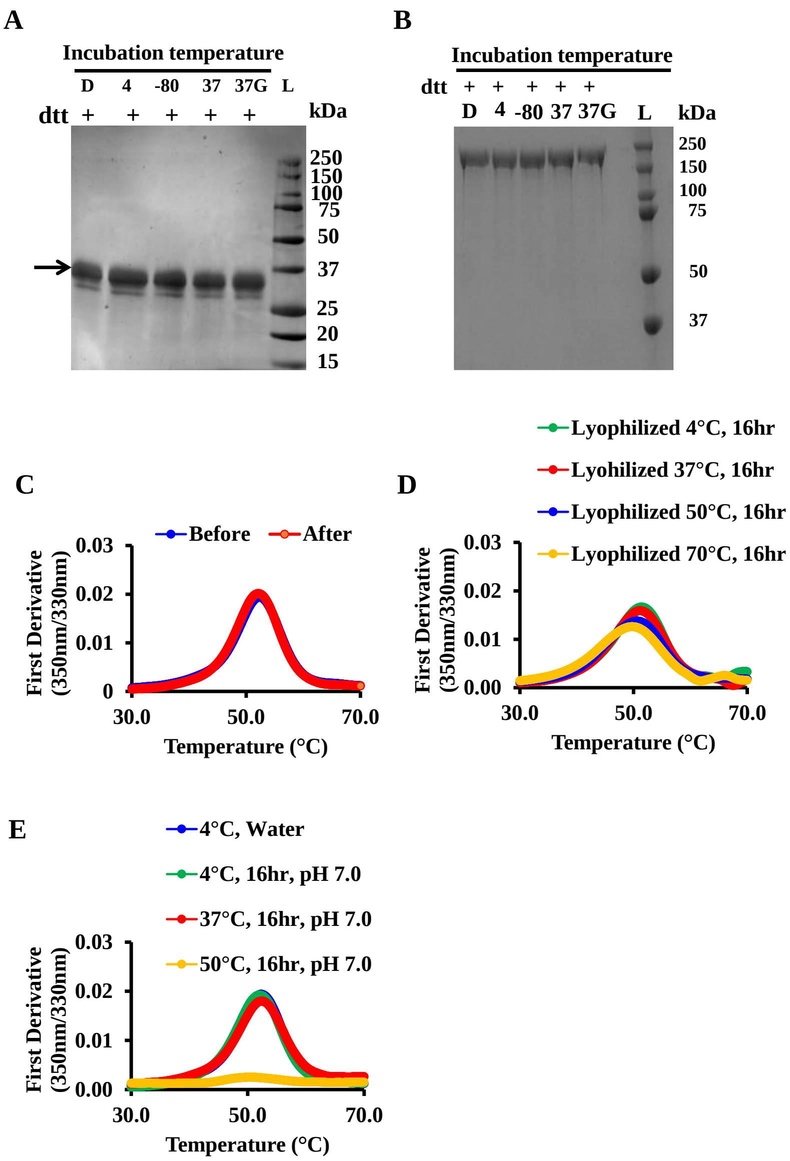

Supplementary Figure 2: Response of purified mRBD and Spike-2P proteins to various stresses. Coomassie stained reducing SDS-PAGE of **A)** mRBD and **B)** Spike-2P after incubation under different conditions: D- Dialysed and stored overnight at 4 °C, 4- 4°C stored protein, -80 °C frozen and thawed protein, 37- protein incubated at 37 °C for 1 hour without glycerol, 37G- protein incubated at 37 °C for 1 hour with 5% glycerol. nanoDSF equilibrium thermal unfolding profiles of **C)** mRBD dialysed against water before (blue) and after (red) lyophilization and resolubilization. **D)** Lyophilized mRBD incubated for 16 hours at 4, 37, 50 and 70 °C and then redissolved in water, prior to nanoDSF. **E)** mRBD in CGH (Citrate,` Glycine, HEPES (1mM each)) buffer, pH 7, incubated for 16 hours at 4°C, 37 °C and 50 °C. The blue trace in E is identical to that in C and inserted to show that the mRBD thermal unfolding profiles in water and CGH buffer, pH7 are very similar.

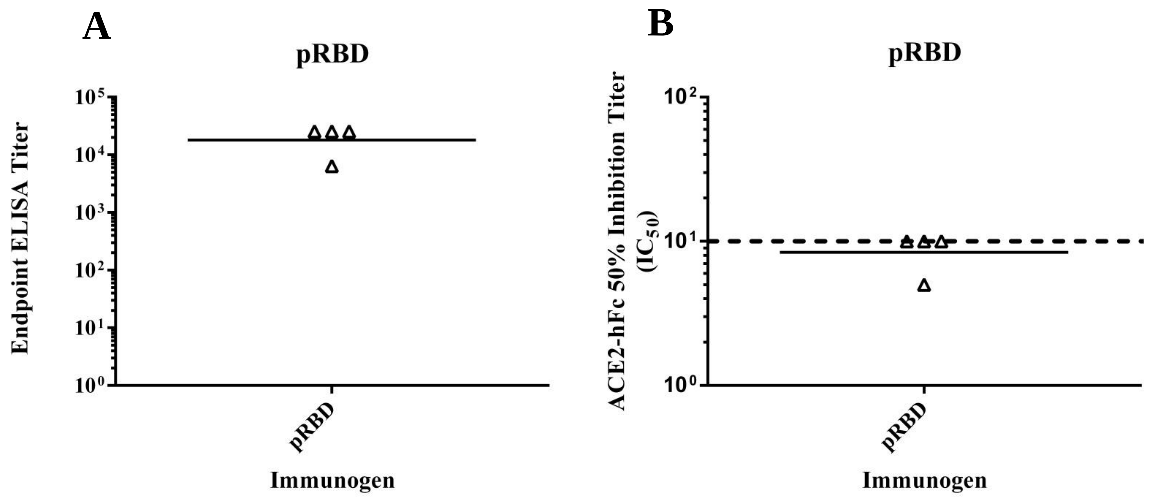

Supplementary Figure 3: Guinea Pig Serum titers obtained after two immunizations with AddaVax™ formulated pRBD. **A)** ELISA endpoint titer against pRBD **B)** 50% Inhibitory titers of ACE2 receptor competing antibodies from guinea pigs immunized with AddaVax™ adjuvanted pRBD. Competition values below 10 are uniformly assigned a value of five. The dashed line represents the value 10.
